# Multidisciplinary team management of congenital dysfibrinogenemia in pregnancy: a case report

**DOI:** 10.1186/s12884-025-07834-3

**Published:** 2025-07-03

**Authors:** Meng Jie He, Zhou Jun Wei, Fei Wang, Hai Ying Zhang

**Affiliations:** 1 Department of Clinical Pharmacy, Sichuan Provincial Women’s and Children’s Hospital, The Affiliated Women’s and Children’s Hospital of Chengdu Medical College, Chengdu, China; 2 Medical Intensive Care Unit, Sichuan Provincial Women’s and Children’s Hospital, The Affiliated Women’s and Children’s Hospital of Chengdu Medical College, Chengdu, 610014 China

**Keywords:** Congenital dysfibrinogenemia, Pregnancy, Amniocentesis, Multidisciplinary team

## Abstract

**Background:**

Congenital dysfibrinogenemia is a rare autosomal dominant disorder involving abnormal fibrinogen function, leading to variable risks of bleeding and thrombosis. Its management during pregnancy is particularly challenging due to the potential for complications such as miscarriage, stillbirth, placental abruption, and fetal growth restriction. The absence of reliable predictive laboratory markers further complicate individualized risk assessment and clinical decision-making.

**Case presentation:**

A 23-year-old primigravida was diagnosed with congenital dysfibrinogenemia after low fibrinogen levels were detected early in her pregnancy. Family history revealed similar fibrinogen abnormalities in her father and brother. Amniocentesis for fetal genetic testing was performed after a multidisciplinary team review that evaluated the bleeding risks, with no complications during or immediately following the procedure. Genetic testing confirmed congenital dysfibrinogenemia in both the mother and fetus. Throughout the pregnancy, the patient was closely monitored, with no signs of bleeding, thrombosis, or other complications. At 39.3 weeks, following a failed attempt at vaginal delivery, she underwent a cesarean section after fibrinogen replacement therapy. The surgery was uneventful, and there were no significant bleeding or thrombotic events postoperatively.

**Conclusions:**

This case emphasizes the critical role of a multidisciplinary team approach in managing congenital dysfibrinogenemia during pregnancy, amniocentesis, and perinatal care. It demonstrates the effectiveness of thorough disease assessment and fibrinogen replacement therapy in preventing bleeding, thereby ensuring good outcomes for both the mother and fetus.

## Background

Fibrinogen plays a central role in hemostasis, contributing to clot formation, platelet aggregation, and fibrinolysis [1, 2]. Reduced fibrinogen levels can result from either hereditary or acquired causes. Hereditary fibrinogen deficiencies are rare, whereas acquired reductions are more commonly associated with conditions such as liver dysfunction, thrombotic thrombocytopenic purpura, hemophagocytic syndrome, and autoimmune diseases. Distinguishing between hereditary and acquired dysfibrinogenemia often involves genetic analysis, family history, and laboratory evaluations, which help guide clinical decisions, particularly in predicting bleeding or thrombotic risks [3].

Hereditary fibrinogen defects can affect either the quantity (hypofibrinogenemia and afibrinogenemia) or the quality (dysfibrinogenemia) of circulating fibrinogen. Pregnant women with congenital dysfibrinogenemia (CD) are at high risk for early pregnancy loss and postpartum hemorrhage (PPH) [4], while those with hypo- or dysfibrinogenemia are also prone to significant bleeding during pregnancy and surgical procedures [1, 2]. In these cases, fibrinogen replacement therapy (FRT) is considered a key component in the management of CD. Fibrinogen concentrate (human) (FCh) has been used effectively for treating fibrinogen disorders during pregnancy [4].

Here, we report the case of a pregnant woman with dysfibrinogenemia who experienced bruising following minor trauma. Managed by a multidisciplinary team (MDT), she underwent amniocentesis without complications, received FRT before a cesarean section, and delivered via cesarean with the application of Hayman sutures—a type of uterine compression suture used to control PPH. No bleeding or thrombotic events occurred during or after the procedure.

## Case presentation

A 23-year-old primigravida with a naturally conceived singleton pregnancy was referred to our center at 27.6 weeks of gestation for further evaluation of persistent hypofibrinogenemia. At her initial prenatal visit at 10 weeks’ gestation at the local hospital, routine coagulation testing revealed markedly reduced fibrinogen levels. The patient reported a history of easy bruising following minor trauma but denied any episodes of spontaneous bleeding. Her menstrual cycles had been regular prior to pregnancy, and no vaginal bleeding was noted during the current gestation. Although no specific treatment was provided locally, coagulation status was closely monitored. Coagulation function tests revealed extremely low fibrinogen levels, with a minimum value of 0.41 g/L (reference range: 2.00–4.00 g/L). Family history revealed fibrinogen abnormalities in the patient’s father and brother during routine checkups. Both were asymptomatic, and no further evaluation or treatment was conducted.

At 27 weeks, she was referred for genetic counseling at our Prenatal Diagnosis Center. The genetics and prenatal diagnosis specialist recommended close monitoring of coagulation function and amniocentesis for family-based exome sequencing. To guide pregnancy management and due to the patient’s strong desire to clarify the genetic basis of her and the fetus’s condition, she consented to undergo amniocentesis and fetal genetic testing.

Upon admission, coagulation tests showed the following: thrombin time (TT) at 37.6 s (reference range: 14.0–21.0 s), fibrinogen (Clauss method) (Fib-Clauss) at 0.79 g/L, fibrinogen (PT-derived method) (Fib-PT-derived) at 2.22 g/L, and a Fib-PT-derived/Clauss ratio greater than 1.43. The prolonged TT and an elevated Fib-PT-derived/Clauss ratio are suggestive of CD. Further investigations revealed normal liver function tests, antinuclear antibody profile, and antiphospholipid antibodies (aPLs). The patient had only taken calcium carbonate supplements, thus excluding liver disease, autoimmune disorders, hematologic conditions, disseminated intravascular coagulation (DIC), and drug-related causes. Additional examinations revealed the D-dimer level was 0.86 µg/mL (reference range: 0–3 µg/mL), which fell within the normal range for mid-pregnancy. Doppler ultrasound of the lower limbs, neck, and abdominal large vessels showed no evidence of thrombosis. Moreover, fetal ultrasound revealed no signs of fetal growth restriction or placental abnormalities.

**Fig. 1:**
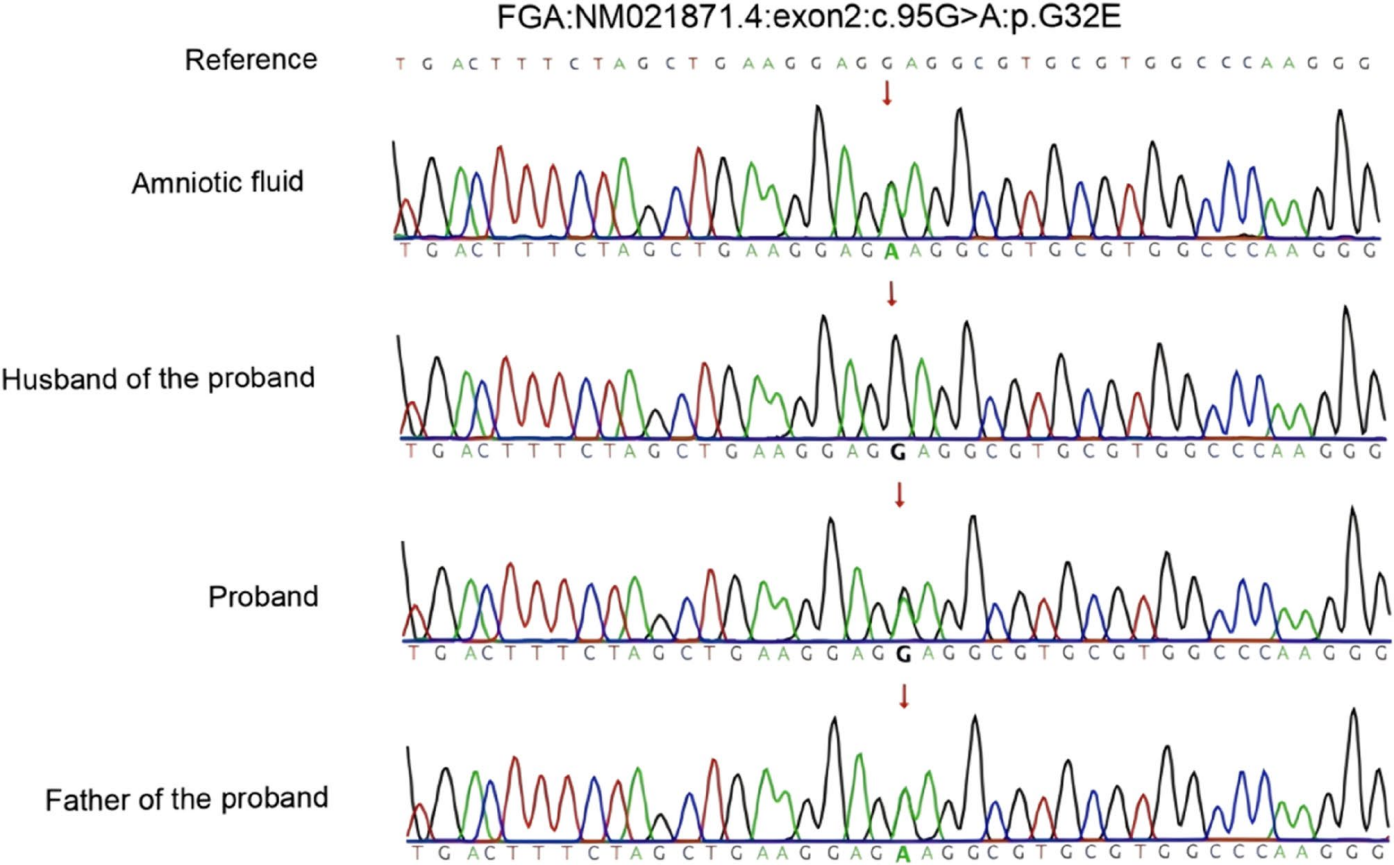
Sanger sequencing results of the mutation site of the FGA gene in the family pedigree

**Fig. 2:**
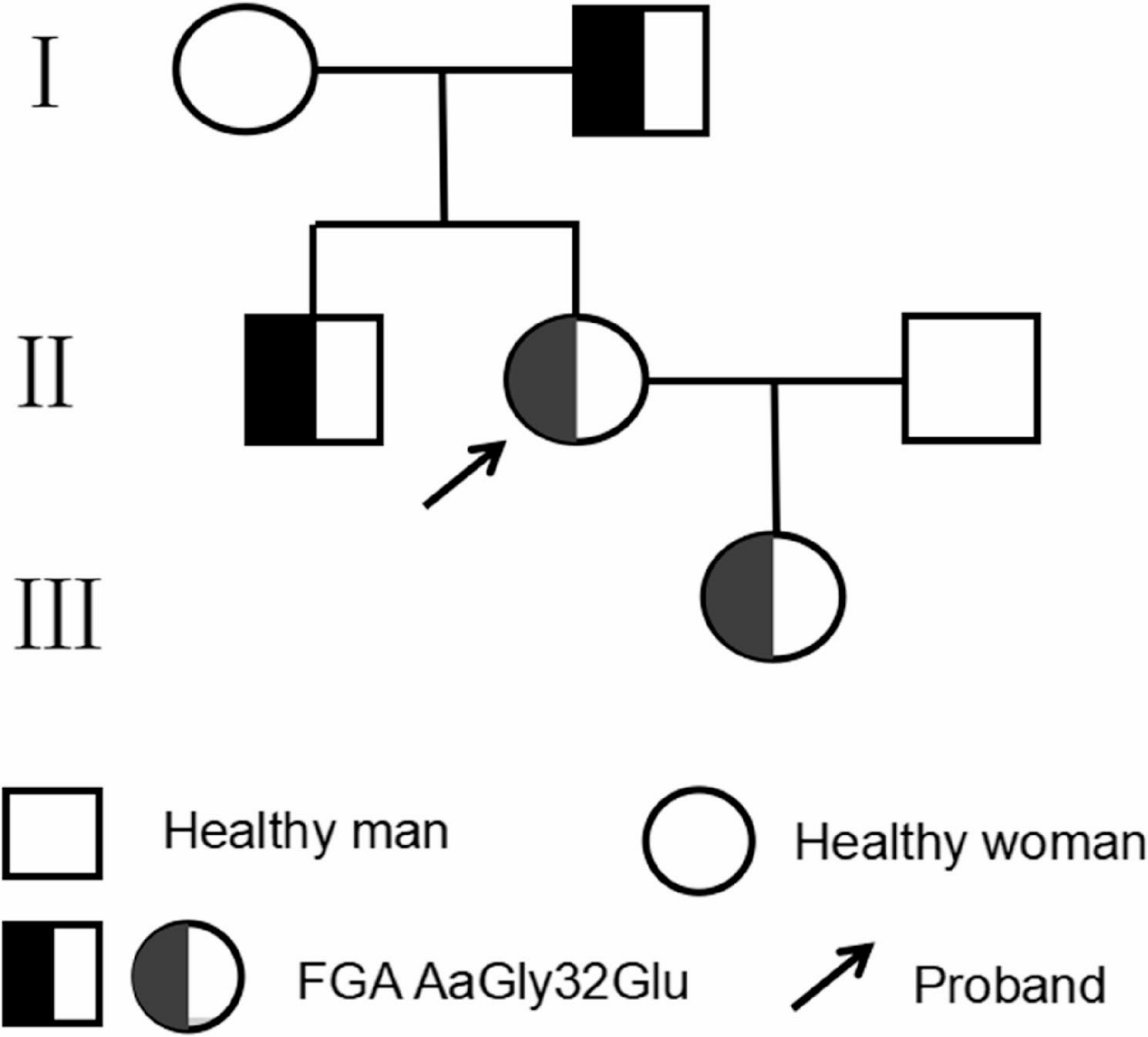
Genealogy of Hereditary Abnormal Fibrinogenemia

Subsequently, a MDT discussion was held to assess the bleeding and thrombotic risks. Considering the low bleeding risk associated with amniocentesis and the patient’s clinical presentation, she was deemed at low risk for bleeding. Amniocentesis was successfully performed on the second day after admission, without the need for FRT before or after the procedure. Postoperatively, only mechanical thromboprophylaxis with intermittent pneumatic compression was applied. The puncture site was closely monitored for any bleeding, and fetal wellbeing was monitored, with no abnormalities detected. The patient was discharged without complications on the second day after the procedure. Following discharge, she continued regular prenatal visits, with monthly monitoring of fetal growth and fibrinogen activity. Genetic testing of the amniotic fluid using high-throughput whole-exome sequencing revealed a heterozygous variant in the FGA gene (NM_021871.4: exon2: c.95G > A: p.G32E). The mutation of glycine at position 32 to glutamate (Gly32Glu) was identified, classified as likely pathogenic according to the American College of Medical Genetics and Genomics (ACMG) standards and guidelines [5]. The sequencing results are shown in Fig. 1, inherited from both the mother and maternal grandfather. The patient’s family pedigree for CD is shown in Fig. 2.

At 38.8 weeks of gestation, the patient was admitted for delivery, with a Fib-Clauss level of 0.79 g/L. Another MDT discussion recommended a trial of vaginal delivery, as there were no medical indications for a cesarean section. Following induction with oxytocin, the patient developed uterine contractions every 1–2 min, each lasting about 30 s, without evidence of cervical dilation. Initial fetal monitoring parameters were within normal limits. Oxytocin administration was discontinued, and tocolytic therapy was initiated to manage uterine tachysystole, resulting in transient symptom relief. After resuming low-dose oxytocin, the cervix dilated to 1 cm over 4 h, and 3 g of FCh was administered. Uterine tachysystole subsequently recurred (every 1–2 min, lasting 40 s), raising concerns about fetal distress or potential placental abruption. In light of these findings, the trial of vaginal delivery was discontinued. At 39.3 weeks, a cesarean section was performed. To minimize the risk of hemorrhage associated with cesarean delivery, 100 µg of carbetocin was administered to enhance uterine contractions. In addition, prophylactic Hayman suturing was performed intraoperatively. The procedure was uneventful, with an estimated blood loss of approximately 300 mL, followed by close monitoring of uterine tone and vaginal bleeding. On postoperative day 3, Fib-Clauss level was rechecked at 1.40 g/L. Therefore, no additional FCh was administered given the long half-life of fibrinogen. Standard thromboembolic risk assessment did not indicate the need for low molecular weight heparin (LMWH). Therefore, mechanical thromboprophylaxis was applied. The patient was discharged on postoperative day 6, with Fib-Clauss level at 1.08 g/L. Placental pathology revealed no thrombus formation or vascular abnormalities.

**Fig. 3:**
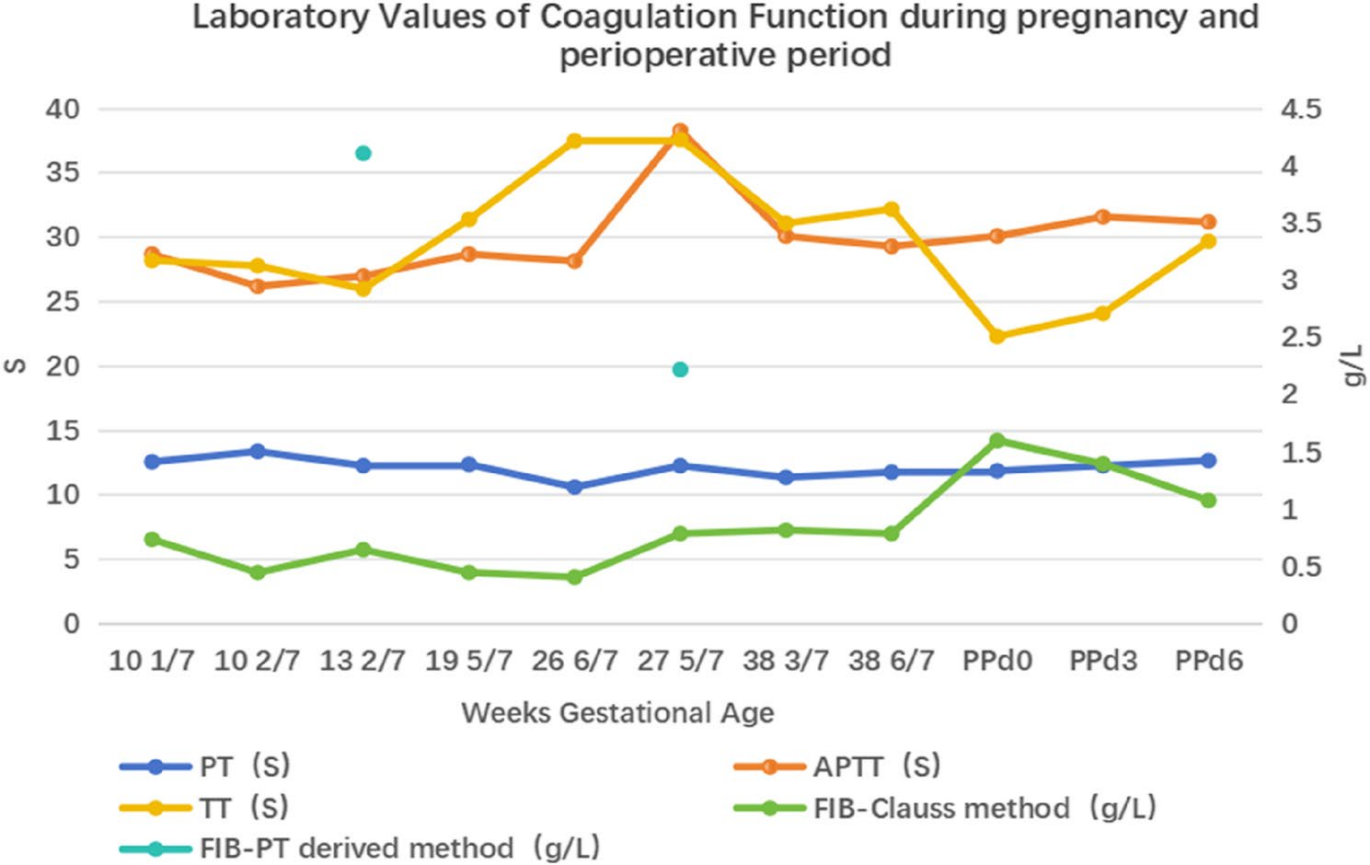
Laboratory Values of Coagulation Function during pregnancy and perioperative period

The patient delivered a healthy female infant (2890 g, 49 cm, Apgar score 10-10-10) with clear amniotic fluid and no bleeding signs. At birth, the infant showed a prolonged TT and a low Fib-Clauss level of 0.33 g/L, consistent with CD. Mild neonatal jaundice developed without bleeding, as confirmed by cranial and abdominal ultrasounds. No bleeding was noted in either the mother or infant at 1-month follow-up, and both remained complication-free at 6 months. Coagulation parameters during pregnancy and the perioperative period are presented in Fig. 3.

## Discussion and conclusions

In this case, the patient’s diagnosis of CD, a rare disorder characterized by impaired fibrinogen function, resulting in abnormal clot formation [6], presented unique clinical challenges, especially in the context of pregnancy. Most patients with CD are asymptomatic, with fibrinogen abnormalities often discovered incidentally during routine physical exams or preoperative screenings [6]. Genetic testing is considered the gold standard for diagnosis, though it is time-consuming and costly. Coagulation function tests, including normal prothrombin time (PT) and activated partial thromboplastin time (APTT), prolonged TT, significantly decreased Fib-Clauss levels, normal or elevated Fib-PT-derived levels, and a Fib-PT-derived/Clauss ratio greater than 1.43, or a Fib-Clauss/PT-derived ratio less than 0.7 [7, 8] are recommended as rapid diagnostic methods in most medical settings. In this case, the patient’s family history and laboratory findings strongly suggested CD, which was confirmed through genetic testing.

CD presents with diverse clinical phenotypes, with only a small proportion of patients exhibiting bleeding or thrombotic complications. Among those with bleeding tendencies, bruising is the most commonly reported manifestation in both European and Chinese populations [9–11]. Women with CD face a higher risk of pregnancy-related complications such as recurrent pregnancy loss, stillbirth, placental abruption, fetal growth restriction, and PPH [11]. Despite this, many patients with CD have uneventful pregnancies, though pregnancy remains a high-risk condition that requires personalized management. Consequently, clinical management during pregnancy and the perinatal period presents significant challenges, necessitating a MDT approach that integrates the patient’s personal history, family history, and prior pregnancy history to formulate an individualized management plan and assess risks of adverse pregnancy events [12]. The patient in this case underwent a multidisciplinary evaluation, with the involvement of obstetricians, Medical Intensive Care Unit (MICU) specialists, haematologists, clinical pharmacists, anesthesiologists and geneticists. The evaluation focused on assessing thrombosis and bleeding risks, as well as managing the pregnancy, amniocentesis, and peripartum period. For patients with a high thrombosis risk, preventive measures should include thromboprophylaxis with LMWH throughout the pregnancy [6]. In Chinese populations, the reported incidence of thrombosis is relatively low, at approximately 3.9% [13, 14].

Given the limited reports on invasive procedures in patients with CD, a MDT planned care for amniocentesis, which represents a particularly novel aspect of this case. A comprehensive risk assessment revealed a low risk of bleeding, which informed the clinical decision. This conclusion was based on: (1) the patient’s mild bleeding phenotype—only minor bruising with no history of mucosal, menstrual, or surgical bleeding; (2) a normal pre-procedural thromboelastography (TEG), which, despite ongoing debate, remains valuable for assessing coagulation status in pregnancy [4, 8, 9, 15]; and (3) evidence showing that vaginal bleeding after amniocentesis occurs in only 2–3% of cases [16], with no significant bleeding risk listed in The Royal College of Obstetricians & Gynaecologists (RCOG) guidelines [17]. These findings supported the MDT’s conclusion of low procedural risk. The amniocentesis was successfully performed without prophylactic hemostatic agents, and the patient’s hemostasis was achieved without complications.

Subsequent whole-exome sequencing identified a pathogenic mutation at the AαGly32Glu site in the FGA gene, confirming the diagnosis of CD. Fibrinogen gene mutations, especially in the Aα chain and thrombin cleavage sites, are strongly linked to thrombosis [6, 18]. These mutations reduce plasminogen binding, impair plasminogen activator activation, and increase self-binding propensity [18]. Notable mutations include AαArg16Cys, AαSer532Cys, γAsp364Val, BβAla68Thr, and AαArg554Cys [6]. In this case, the identified mutation has not been linked to increased risk of thrombosdata generated or analysed during this study are included in this published artis. Accordingly, the case was classified as type 3 A, which is characterized by a low thrombotic risk. The classification of dysfibrinogenemia is summarized in Table 1 [19].

**Table 1:** Classification of dysfibrinogenemia

Type	Description	ThrombosisRisk
Type 3A	Dysfibrinogenemic patients either with bleeding phenotype or with thrombotic phenotype not fulfilling criteria for dysfibrinogenemia 3B or asymptomatic individuals	Low
Type 3B	Dysfibrinogenemic patients carriers of a thrombotic fibrinogen mutation or suffering from thrombotic events with a first-degree familial thrombotic history (relatives with the same genotype) without any other thrombophilia	High

Given the elevated risk of obstetric complications in CD, attributable to fibrinogen’s critical role in maintaining fetal–maternal circulation and peripartum hemostasis, the patient was placed under MDT management to ensure individualized care tailored to her risk profile during pregnancy [6, 20]. FRT may be warranted in cases of recurrent pregnancy loss or placental insufficiency to maintain fibrinogen levels ≥ 1 g/L [6], and it is considered for potential vaginal bleeding with a target fibrinogen level ≥ 1.5 g/L [21]. In contrast, routine FRT or anticoagulation is generally unnecessary in uncomplicated pregnancies, where regular monitoring suffices. In this case, the pregnancy was uneventful apart from mild bruising. Although thrombosis-related mutations have been associated with fetal growth restriction and placental abruption [19], this patient, classified as low thrombotic risk, showed normal fetal growth throughout gestation.

The timing of delivery was determined based on a comprehensive assessment of both maternal and fetal conditions. Following MDT discussion and obstetric evaluation, a favorable pregnancy outcome was anticipated, and vaginal delivery was initially recommended. Upon admission, the patient’s Fib-Clauss level was 0.79 g/L. Given that natural labor, regional anesthesia, and cesarean section require maintaining fibrinogen ≥ 1.5 g/L [19], FRT was considered necessary to optimize peripartum hemostasis. The clinical pharmacist recommended FCh due to its pharmacokinetics, achieving peak fibrinogen levels within 2 h and improving clot firmness 1 h post-infusion [22]. However, FCh infusion may increase the risk of pulmonary embolism [23] or deep vein thrombosis [24] in patients with CD. Therefore, FCh was given after labor onset, and at least 2 h before cesarean section. The dose was calculated based on the patient’s pregnancy weight [25]. Good communication with the blood bank ensured sufficient availability of blood products for management of PPH. Oxytocin was given to induce labor, and 3 g of FCh was administered once labor onset was confirmed. Due to frequent, intense contractions raising concerns of fetal distress or placental abruption, the vaginal delivery plan was abandoned, and a cesarean section was performed 3 h later [25]. 2 h after infusion, the fibrinogen level was 1.60 g/L, and epidural anesthesia was chosen in consultation with the anesthesiology department. Despite no significant bleeding during pregnancy, patients with CD are still at higher risk for PPH. Carbetocin and intraoperative Hayman suturing were used to manage uterine atony and prevent bleeding. Given fibrinogen’s half-life of 3–4 days, levels returned to pre-infusion values by day 9 [22]. Postoperatively, the patient experienced no significant bleeding, and no additional FCh was given. Fibrinogen levels on postoperative days 3 and 6 were 1.40 g/L and 1.08 g/L, respectively, which were consistent with findings reported by Ross et al. [23] and Mumford et al. [25]. A postoperative thrombosis risk assessment was performed according to standard protocols for pregnant women. Her postpartum venous thromboembolism (VTE) risk score was 2 (2 points for conversion to cesarean section). As her score was below 3, only mechanical prophylaxis was administered. The postoperative course was uneventful, with favorable maternal and neonatal outcomes and no observed complications.

In conclusion, this case underscores the importance of personalized, multidisciplinary management in pregnant patients with CD. Moreover, this case reinforces the public health value of early diagnosis and genetic counseling in hereditary bleeding disorders, emphasizing the need for national screening programs and the incorporation of multidisciplinary strategies into obstetric care for rare coagulation conditions.

This case highlights promising avenues for future research, including risk stratification through personalized biomarkers, optimization of FRT protocols, and the development of procedural guidelines for CD management in pregnancy, particularly within the context of MDT-led care.

## Data Availability

All data generated or analysed during this study are included in this published article.

## Abbreviations

ACMG: American College of Medical Genetics and Genomics
aPLs: Antiphospholipid antibodies
APTT: Activated partial thromboplastin time
CD: Congenital dysfibrinogenemia
DIC: Disseminated intravascular coagulation
FCh: Fibrinogen concentrate (human)
Fib-Clauss: fibrinogen (Clauss method)
Fib-PT-derived: fibrinogen (PT-derived method)
FRT: Fibrinogen replacement therapy
LMWH: Low molecular weight heparin
MDT: Multidisciplinary team
MICU: Medical Intensive Care Unit
PPH: Postpartum hemorrhage
PT: Prothrombin time
RCOG: Royal College of Obstetricians & Gynaecologists
TEG: Thromboelastography
TT: Thrombin time
VTE: Venous thromboembolism
